# Initiating Discussion of Care Partner Mental Health During Clinical Encounters for Persons with Dementia

**DOI:** 10.1093/geroni/igaf122.4293

**Published:** 2025-12-31

**Authors:** Lisa Mistler, Kady Sternberg, Alejandra Martinez, Joshua Chodosh, Paul Barr

**Affiliations:** Geisel School of Medicine, Lebanon, New Hampshire, United States; Dartmouth-Hitchcock Medical Center, Lebanon, New Hampshire, United States; The Dartmouth Institute for Health Policy and Clinical Practice, Lebanon, New Hampshire, United States; NYU Langone, New York City, New York, United States; The Dartmouth Institute for Health Policy and Clinical Practice, Lebanon, New Hampshire, United States

## Abstract

**Objectives:**

Care partners (CP) of persons with dementia (PwD) experience considerably higher depression rates than non-CP peers. Current guidelines provide no strategies for implementing recommended mental health assessments for CP, whose interaction with clinicians is primarily through visits accompanying PwD. The objective of this study is to assess CP mental health support by clinicians of PwD during routine office visits for PwD.

**Methods:**

Data comprise transcripts of 200 initial clinical visits of PwD accompanied by CP. Two IRB-approved study team members independently analyzed 10 randomly-selected transcripts from urban clinics and 10 from rural clinics. Quantitative analysis included number of visits, number of times CP mental health was mentioned, who initiated the topic and the response to broaching the topic. Qualitative content analysis identified themes and patterns of clinician, CP, and PwD responses and mental health topics discussed.

**Results:**

In our preliminary analysis of 20 transcripts, CP mental health was mentioned in 10% of urban and 30% of rural visits. Four patterns were identified: 1) clinician inquired, unprompted, about CP mental health and CP responded; 2) CP reported mental health concerns and clinician did not respond; 3) CP reported mental health concerns and clinician responded; 4) CP mental health not discussed.

**Implications:**

Preliminary data indicate a difference in frequency of and approaches to assessing CP mental health during PwD clinician visits in rural versus urban clinics. Knowing factors associated with clinicians asking about CP mental health will help to develop tailored interventions for screening and referring CP for mental health care.

